# Crossover of the Hypothalamic Pituitary–Adrenal/Interrenal, –Thyroid, and –Gonadal Axes in Testicular Development

**DOI:** 10.3389/fendo.2014.00139

**Published:** 2014-08-27

**Authors:** Diana C. Castañeda Cortés, Valerie S. Langlois, Juan I. Fernandino

**Affiliations:** ^1^Laboratorio de Biología del Desarrollo, Instituto de Investigaciones Biotecnológicas, Instituto Tecnológico de Chascomús, Universidad Nacional de San Martín y Consejo Nacional de Investigaciones Científicas y Técnicas, Chascomús, Argentina; ^2^Chemistry and Chemical Engineering Department, Royal Military College of Canada, Kingston, ON, Canada

**Keywords:** thyroid hormone, corticotropin-releasing hormone, gonadotropins, androgen, testis, fish, amphibians

## Abstract

Besides the well-known function of thyroid hormones (THs) for regulating metabolism, it has recently been discovered that THs are also involved in testicular development in mammalian and non-mammalian species. THs, in combination with follicle stimulating hormone, lead to androgen synthesis in *Danio rerio*, which results in the onset of spermatogenesis in the testis, potentially relating the hypothalamic–pituitary–thyroid (HPT) gland to the hypothalamic–pituitary–gonadal (HPG) axes. Furthermore, studies in non-mammalian species have suggested that by stimulating the thyroid-stimulating hormone (TSH), THs can be induced by corticotropin-releasing hormone. This suggests that the hypothalamic–pituitary–adrenal/interrenal gland (HPA) axis might influence the HPT axis. Additionally, it was shown that hormones pertaining to both HPT and HPA could also influence the HPG endocrine axis. For example, high levels of androgens were observed in the testis in *Odonthestes bonariensis* during a period of stress-induced sex-determination, which suggests that stress hormones influence the gonadal fate toward masculinization. Thus, this review highlights the hormonal interactions observed between the HPT, HPA, and HPG axes using a comparative approach in order to better understand how these endocrine systems could interact with each other to influence the development of testes.

## Introduction

Thyroid hormones (THs) have been implicated in a plethora of physiologic actions, such as metabolism, development, growth, and reproduction [reviewed in Ref. (1–5)]. In the last years, the influence of THs in gonadal development has been intensively studied in rodent species (2, 6–10); however, data remains scarce on the roles of THs in non-mammalian reproduction [reviewed in Ref. (2, 6–12)]. As endocrine axes are well conserved among vertebrates, a comparative approach to review TH function and regulation in gonadal development would help to better understand non-mammalian endocrine systems. Thus, this paper provides a comprehensive review of existing literature on the effects of THs in testicular development in non-mammalian species, highlights the interaction of the hypothalamic–pituitary–thyroid (HPT) gland, –adrenal/interrenal (HPA), and –gonadal (HPG) axes (Table 1), and identifies key areas for future research.

**Table 1:** **Summary of studies that shows the interaction between the hypothalamic–pituitary–adrenal/interrenal and thyroid gland axes (HPA–HPT), –adrenal/interrenal and –gonadal axes (HPA–HPG), and –thyroid gland and –gonadal axes (HPT–HPG)**.

Species	Treatment	Response	Reference
**HPA–HPT**			
**Fish**
*Oncorhynchus kisutch*	*In vitro* CRH	↑TSH	Larsen et al. (13)
**Amphibians**
*Rana catesbeiana*	*In vitro* CRH	↑TSH	Ito et al. (14), Kaneko et al. (15)
	*In vitro* antisauvagine-30	↓TSH	Okada et al. (16)
*Rana pipiens*	*In vitro* ovine CRH	↑TSH	Denver (17)
*Xenopus laevis*	*In vivo* and *in vitro Xenopus* CRH	↑T4, TSH	Boorse and Denver (18)
**Reptiles**
*Pyrgulina scripta*	*In vitro* Ovine CRH	↑TSH	Denver and Licht (19, 20)
**Birds**
*Gallus gallus*	*In vivo* ovine CRH	↑T4, T3	Meeuwis et al. (21)
	*In vivo* ovine CRH	↑T4, T3, TSH	Geris et al. (22)
	*In vitro* CRH-R2	↑TSH	De Groef et al. (23)
**HPA–HPG**			
**Fish**
*Odontesthes bonariensis*	*In vitro* cortisol	Masculinization	Fernandino et al. (24), Hattori et al. (25)
		↑11-KT, *ar*	
		↓*cyp19a1a*	
*Paralichthys olivaceus*	High temperature and cortisol	Masculinization	Yamaguchi and Kitano (26)
	*In vitro* cortisol	↓*cyp19a1*	Yamaguchi et al. (27)
*Oryzias latipes*	High temperature	Masculinization	Hayashi et al. (28)
		High cortisol levels	
*Pseudocrenilabrus multicolor victoriae*	Hipoxia	↑T, male-based sex ratio	Friesen et al. (29)
*Oreochromis niloticus*	High temperature	↓*cyp19a1a*, masculinization	Baroiller et al. (30)
*Oncorhynchus mykiss*	*In vitro* cortisol	↑11-KT	Shulz (31)
**Mammals**
*Cavia aperea*	Early social stress	Masculinization	Kaiser et al. (32)
**HPT–HPG**			
**Fish**
*Verasper moseri*	*In vivo*, *in vitro* sbGnRH	↑T4	Chiba et al. (33)
*Oncorhynchus masou*	
*Carassius auratus*	
*Channa gachua*	*In vivo* GnRH	↑T4	Roy et al. (34)
*Catla catla*	
*Carassius auratus*	*In vivo* GnRH	↑T4	MacKenzie et al. (35)
	*In vivo*, *in vitro* T3	↓*cyp19a*	Nelson et al. (36)
*Salmo-gairdneri Richardson*	*In vivo* Testosterone	↓T3	Leatherland et al. (37)
*Danio rerio*	*In vivo* T3	↑Proliferation	Morais et al. (38)
		Sertoli cells	
		↑Proliferation type A spermatogonia	
	*In vitro* TH + FSH	↑11-KT	
*Clarias gariepinus*	*In vitro* thiourea	↓11-KT	Swapna et al. (39)
	*In vivo* thiourea	↓*11ß-hsd*, *11ß-h*, ↑*cyp19a1*	Rasheeda et al. (40)
*Oreochromis niloticus*	*In vivo* T3	↑GnRH cells	Parhar et al. (41)
*Anabas testudineus*	*In vitro* T3	↑3ß-hsd	Nagendra Prasad et al. (42)
**Amphibians**
*Rana catesbeiana*	*In vitro* mGnRH	↑TSH, T4	Denver (17)
*Ambystoma mexicanum*	*In vivo* LHRH	↑T4	Jacobs and Kuhn (43)
*Rana ridibunda*			Jacobs et al. (44)
*Rana temporaria*	
*Rana escuelita*	
*Rana pipiens*	*In vitro* mGnRH	↑TSH	Okada et al. (45)
	*In vivo* T3	↓*cyp19*	Hogan et al. (46)
*Physalaemus pustulosus*	*In vivo* T3	↑*ar*, ↓*cyp19*, ↓*srd5a1*	Duarte-Guterman et al. (47)
*Silurana tropicalis*	*In vivo* T3	↑*ar*, *srd5a1, srd5a2*	Duarte-Guterman and Trudeau (48)
	*In vivo*, potassium perchlorate	↑*srd5a2*, ↓*ar*	Flood and Langlois (151)
*Lithobates sylvaticus*	*In vivo*, sodium perchlorate	↓*cyp19*	Duarte-Guterman et al. (49)
**Reptiles**
*Podarcis sicula*	*In vivo* T3	↑*ar*	Cardone et al. (50)
**Birds**
*Gallus gallus*	*In vivo* T3	↓LH	Jacquet et al. (51)
	*In vivo* propylthiouracil	↑T	Akhlaghi and Zamiri (52)
	*In vivo* T3	↓*cyp19*	Sechman (53)
*Coturnix japonica*	*In vivo* thiourea	↓T	Weng et al. (54)
**Mammals**
*Rattus norvegicus*	*In vitro* T3, T3 + FSH	↑*Ar*	Arambepola et al. (55)
	*In vitro* T3	↑AR	Panno et al. (56)
	*In vivo* T3	↑Proliferation	Marchlewska et al. (57)
		Sertoli cells	
		↑Proliferation	
		Germ cells	
	*In vitro* T3	↓CYP19	Ulisse et al. (58)
		↓CYP19	Andò et al. (59)
		↓CYP19, *Cyp19*	Pezzi et al. (60)
	*In vivo* propylthiouracil	↑*Cyp19*	Hapon et al. (61)
	Thyroidectomy	↓*3ß-Hsd*, *17ß*-*Hsd*	Antony et al. (62)
		↓*17ß-Hsd*	Biswas et al. (63)
	*In vivo* T4	↑SDR5*a*	Kala et al. (64)
		↑*Srd5a*	Ram and Waxman (65)
	Methimazole	↓*Srd5a1*, *Srd5a2*	Anbalagan et al. (66)
	Hypothyroid conditions	↓LH	Romano et al. (67)
	Propylthiouracil	↓T	Chiao et al. (68)
	*In vivo* methimazole	↓LH	Valle et al. (69)
	*In vivo* 2,8-Dimercapto-6-hydroxypurine	↓T	Jahan et al. (70)
	*In vivo* hypothyroid conditions	↑GnRH; ↓T, LH	Maran et al. (2), Wagner et al. (8)
	*In vivo* T4 thyroidectomy	↑GnRH; ↓T, LH	Chiao et al. (71)
*Mus musculus*	*In vitro* T3	↓CYP19, *Cyp19*	Catalano et al. (72)
		↑C*yp17*	Manna et al. (73)
	*In vitro* T3 + FSH	↓CYP19, *Cyp19*	Cecconi et al. (74)
*Sus scrofa domestica*	*In vitro* T4, T3	FSH-induced aromatase activity	Chan and Tan (75)
	*In vitro* T3	↓CYP19	Gregoraszczuk et al. (76)
*Ovis aries*	Thyroidectomy	↑FSH	Anderson et al. (77)

*An upward pointing arrow indicates an increase in gene expression, hormone concentration, or enzyme activity; whereas a downward pointing arrow indicates a decrease*.

## Hypothalamic Regulation of THs

**Figure 1:**
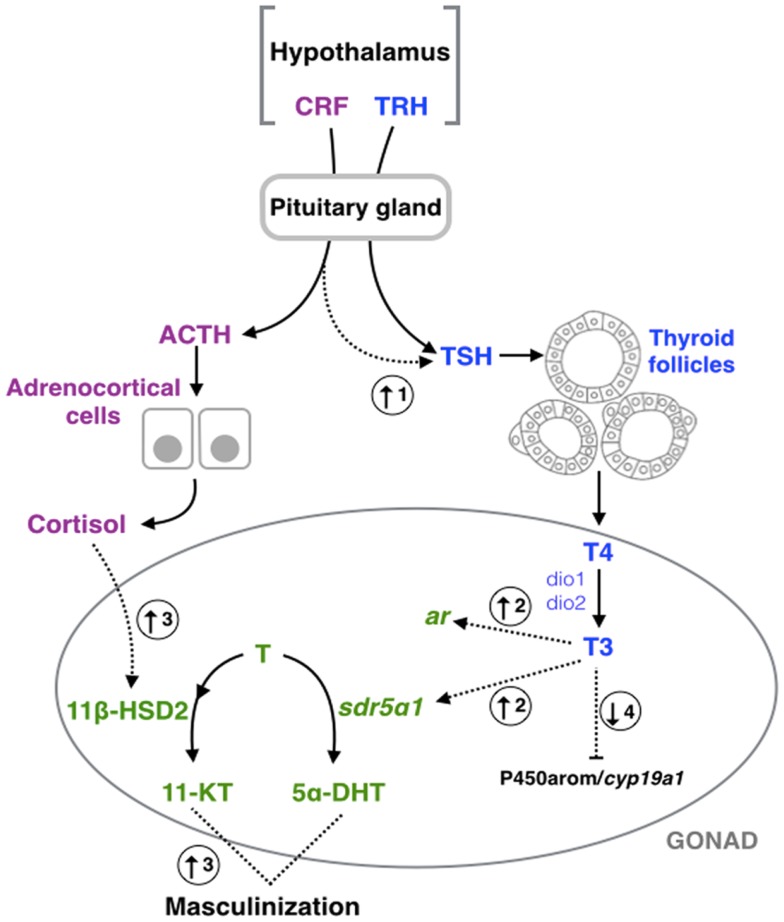
**Schematic representation of hypothalamic–pituitary– thyroid gland (blue), –adrenal/interrenal (purple), and –gonadal interactions (green)**. Dashed arrows represent the points of interaction between the different axes highlighted in this review. (1) Corticotropin- releasing factor (CRF) could induce the pituitary–thyroid stimulating hormone (TSH) secretion in fish (13), amphibians (15, 16, 82), and birds (23). (2) Triiodothyronine (T3) could increase the expression of type a1 steroid 5-alpha-reductase type 1 (*sdr5*α*1*) and androgen receptor (*ar*) in amphibians (83). (3) Exposure to cortisol results in an increase of the androgen-related machinery and subsequent masculinization in fish (25–27, 84), and mammals (32). (4) Exposition of thyroid hormones could inhibit the aromatase (P450arom/*cyp19a1*) activity or expression in fish (36, 85), amphibians (46, 47), and mammals (58, 59, 75). TRH, thyrotropin-releasing hormone; Dio1, deiodinase type 1; Dio2, deiodinase type 2; T4, thyroxine; ACTH, pituitary adrenocorticotropic hormone; T (testosterone) 11β-HSD2, type 2 isozyme of 11β-hydroxysteroid dehydrogenase; 11-KT, 11-ketotestosterone; 5α-DHT, 5α-dihydrotestosterone.

The central nervous system (CNS) is stimulated by environmental factors to regulate TH homeostasis. Thus, the hypothalamic tripeptide thyrotropin-releasing hormone (TRH) stimulates the anterior pituitary to synthesize and secrete the thyroid-stimulating hormone (TSH; Figure 1). The action of TRH has been confirmed in tetrapods [reviewed in Ref. (78, 79)]; however, in fish, mixed effects have been found. In bighead carp (*Aristichthys nobilis*) and Japanese eel (*Anguilla japonica*), TRH was shown to increase hypophyseal *tsh-*β expression (80, 81), while in coho salmon (*Oncorhynchus kisutch*), TRH-treatment did not stimulate TSH release (13). Furthermore, teleost fish have no portal systems that connect the CNS and the pituitary, in which hypothalamic neurons terminate very close to adenohypophysial cells (79). These findings suggest that TRH is not a major TSH-releasing factor in fish.

In addition to TH regulation, it has been suggested that HPT is also involved with the HPA axis [*O. kisutch*, *Rana catesbeiana*, *Rana pipiens*, *Xenopus laevis*, *Pyrgulina scripta*, *Gallus gallus* (see Table 1)]. It is well known that the corticotropin-releasing hormone (CRH, also known as the corticotropin-releasing factor or CRF) is a potent stimulator of the pituitary adrenocorticotropic hormone (ACTH), which stimulates the synthesis and secretion of cortisol, the main stress hormone in vertebrates (86–88). A decade ago, De Groef et al. (23) observed that CRH can induce pituitary TSH secretion in chicken (*G. gallus*) through the CRH type 2 receptor (CRH-R2) expressed on pituitary thyrotrope cells, linking both of these endocrine axes (Figure 1). Similar results have been observed in fish, amphibians, reptiles, and other bird species [Table 1; reviewed in Ref. (13, 82, 89–91)]. The dual hypophysiotropic action of CRH has several effects on the peripheral hormonal function of the HPT axis. In amphibians, metamorphosis is dependent on THs; however, changes in CRH molecular machinery have been observed during this period of development. For example, the expression of both *crh* and *crh-r2* increase significantly throughout frog metamorphosis (92). Noteworthy, *crh* transcripts start being detected earlier than *crh-r2*, i.e., during premetamorphosis, while the expression of *crh-r2* only begins to be detected later during prometamorphosis (92). Furthermore, it has been observed that treatment with corticosteroids synergizes with THs, leading to an accelerated metamorphosis (93). Thus, Denver (91) hypothesized that both CRH and corticosteroids act on THs in order that tadpoles may respond quickly to environmental cues early in development and metamorphose according to their environment. This crosstalk between HPA and HPT allows frogs to escape from and survive in habitat desiccation and crowding, or food restriction during mid- to late prometamorphosis (91). Similar to fish, CRH-like peptide treatment lead to a significant concentration-dependent increase in TSH secretion of salmonids pituitary culture (13, 94). During smoltification of Atlantic salmon (*Salmo salar*), a critical period of midlife transition from freshwater to seawater with morphological, physiological, and behavioral modifications (95), the increase in THs induced a positive-feedback in the maturation of the CRF neurons [CRF neurogenesis; (96)]. Also, during early development of fish, chronological correlation between ACTH and TSH production has been observed in the pituitary of European sea bass (*Dicentrarchus labrax*) larvae (97). Together, this data suggest that stressor-challenge drives the THs to play both fundamental and modulatory roles in the stress response [reviewed in Ref. (89, 90)]. Moreover, a reduction in basal plasma cortisol levels was observed in hyperthyroidism-induced *Cyprinus carpio* (98). Thus, from the crosstalk between HPA and HPT axes, three main observations can be deduced: (i) CRH acts as a common neuroregulator of the thyroidal and adrenal/interrenal axes in non-mammalian species; (ii) the HPA and HPT axes perform concerted actions on energy metabolism and development; and (iii) the regulation, inhibition, or stimulation of CRH on the TH axis could be dependent on both stage of life and the nature of the tissues being analyzed.

## TH Regulation by Gonadotropins

The HPG axis controls signaling and biosynthesis by the sex steroids. The hypothalamic peptide gonadotropin-releasing hormone (GnRH) regulates the biosynthesis and secretion of both gonadotropins; luteinizing hormone (LH) and follicle stimulating hormone (FSH). Besides the well-known function of GnRH in regulating gonadotropins, GnRH treatment has been shown to moderately increase TSH secretion in amphibians (17, 45), suggesting that GnRH can modulate THs at the pituitary level. Several studies have also observed that GnRH can increase thyroxine (3,5,3′,5′-l-tetra-iodothyronine or T4) levels in fish (33, 34) and in amphibians (44, 99). However, no changes in triiodothyronine (3, 3′, 5-triiodo-l-thyronine or T3) concentrations were observed in plasma after injections of a superactive analog of GnRH in goldfish [*Carassius auratus*; (35)]. Thus, additional work should investigate the possible targets of GnRH in the TH axis.

**Figure 2:**
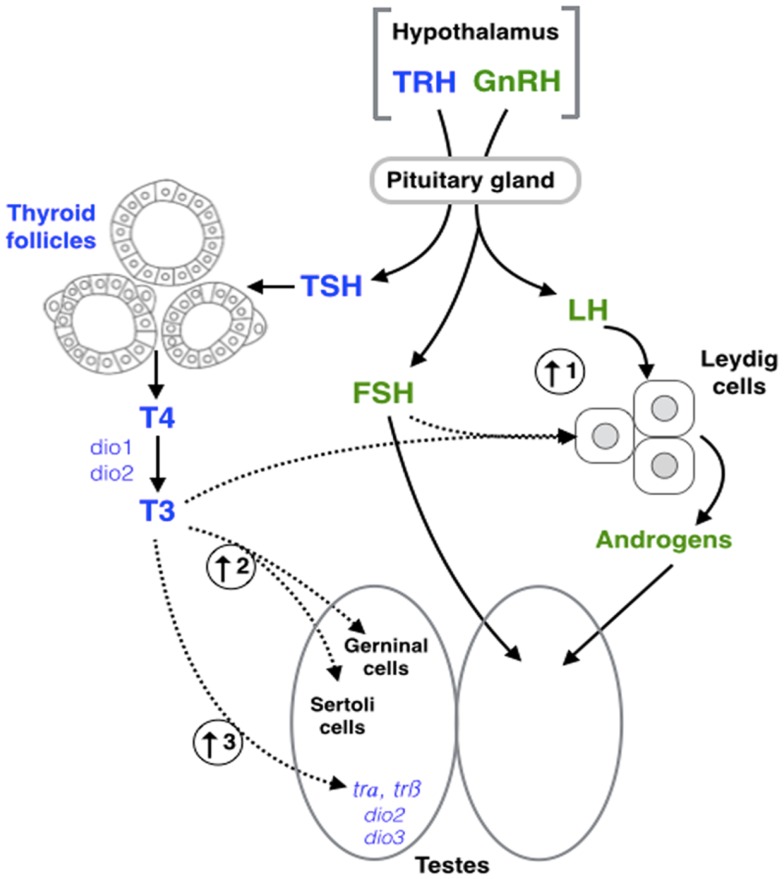
**Schematic representation of hypothalamic–pituitary– thyroid (blue) and gonadal (green) axes interaction**. Dashed arrows represent the points of interaction between the different axes highlighted in this review. (1) Triiodothyronine (T3) in combination with follicle stimulating hormone (FSH) increase 11-ketotestosterone (11-KT) synthesis in fish (38). (2) T3 exposure results in an increase of Sertoli and germ cell (GC) proliferation in fish (38), and mammals (57). (3) T3 increases the expression of thyroid receptor α (*tr*α), thyroid receptor β (*trβ*), deiodinase type 2 (*dio2*), and deiodinase type 3 (*dio3*) in amphibians (83). GnRH, gonadotropin- releasing hormone; LH, luteinizing hormone; TRH, thyrotropin-releasing hormone; TSH, thyroid-stimulating hormone; T4, thyroxine; Dio1, deiodinase type 1.

Luteinizing hormone and FSH are the main regulators of various physiological processes related to formation and maintenance of the gonadal structures (12, 100). In males, FSH is involved in the paracrine control and the structural and nutritional support of germ cell development of the Sertoli cells, while LH regulates androgen production in the Leydig cells (101, 102). The level of both gonadotropins, as well as related gene expression, can be altered by hyper- and hypothyroidic conditions in *Mus musculus* (8, 9, 68, 71). Moreover, studies have shown that THs can interfere with the regulatory activity of FSH, influencing the rate of proliferation and the functioning of Sertoli cells of *Rattus norvegicus* (57, 103) and *Danio rerio* (38). The Sertoli cells are found within the seminiferous tubules and are responsible for spermatogenesis (104). The initiation of spermatogenesis requires several hormones, including FSH and androgens (105–107). For example, thyroidectomized rams (*Ovis aries*) – during their seasonal testicular regression – show an increase in blood FSH concentration and a faster testis growth (77, 108). Moreover, in a testis tissue culture of *D. rerio*, T3 in combination with FSH increases 11-ketotestosterone (11-KT) synthesis (38); the main bioactive androgen in fish (Figure 2). Thus, it has been proposed that FSH partially mediates the effects of THs in male sexual development in *D. rerio*.

Fluctuations in circulating TH levels lead to subsequent changes in the synthesis, secretion, circulation levels, metabolism, and physiological action of androgens. LH induces steroidogenesis in the Leydig cells, which are responsible for the production of androgens. Like FSH, the biosynthesis of LH is subject to the influence of THs. Hypothyroid conditions decrease circulating LH concentrations or LH bioactivity in several vertebrates [e.g., cockerel [*G. gallus*; (51)], rat [*R. norvegicus*; (67)]] as well as the level of testosterone (T) [e.g., *R. norvegicus*; (67, 68, 71)]. Similarly, severe hypothyroidism in *R. norvegicus* decreases proliferation of Leydig cells (109) and increases morphology alterations in the human testes (4, 110). Together, these studies demonstrate that fluctuations in THs can directly modulate gonadotropin actions and provide an indirect mechanism of action in which THs can impact Leydig cell proliferation, androgen biosynthesis, and ultimately, spermatogenesis. The crosstalk between both gonadotropins and THs suggests the existence of a vertebrate-wide interaction between the HPT and HPG axes.

## TH-Related Machinery within Gonadal Tissues

Distribution of TH-related machinery in gonadal tissue is highly sex-specific. TSH stimulates the thyroid gland to synthetize and secrete T4, which is mainly converted into T3 by different types of deiodinases [Dios; (111–113)]. Thus, deiodinases (type 1, 2, and 3) play a major role in achieving the levels of intracellular T3 in target tissues by the deiodination of T4. Deiodinases have been identified in the testes of vertebrate species [e.g., rainbow trout, *Oncorhynchus mykiss* (114), Western clawed frog, *Silurana tropicalis* (48), *G. gallus* (115), and *R. norvegicus* (116)]. The roles of deiodinases in the mammalian testis have been reviewed in detail (9). In developing *R. norvegicus*, the activity of Dio1 and Dio2 is higher in the testes than in the ovaries, whereas Dio3 activity is greater in the ovary tissue (116). Moreover, deiodinase activity (Dio1, Dio2, and Dio3) is predominant during developmental periods (neonatal and weanling), and subsequently declines in the adult life of *R. norvegicus* (116). Similar observations have been confirmed in teleosts. For example, testes of striped parrotfish (*Scarus iseri*) are characterized by higher levels of *dio2* and *dio3* mRNA than in ovaries (117). The transcripts encoding *dio2* mRNA in *O. mykiss* reach their highest levels in the testes during stage II (beginning of spermatogenesis); a period characterized by the differentiation of somatic testicular cells, active proliferation of spermatogonia, and the formation of spermatocysts. At this point, *dio2* expression progressively decreases to later stages of spermatogenesis (114). These results support the idea that TH availability is highly regulated in testicular development and during spermatogenesis by deiodinase activity.

Other important components of the HPT axis are the thyroid receptors (TRs). THs mediate TR signaling and are crucial for testis development and function. The expression of *trs* in testicular tissue and the physiological implications in mammalian species have been reviewed thoroughly (118, 119). Thus, *tr*α and *tr*β code for a number of *tr*-isoforms, including: *tr*α*1*, *tr*α*2, tr*α*3*, *tr*β*1*, *tr*β*2*, and *tr*β*3*, which have been identified in the testes of several vertebrates: fish (114, 117), amphibians (47, 83, 120), reptiles (50), and mammals (104, 121–125). In all vertebrate classes, TRs have been localized in Sertoli cells indicating that this cell-type is an evolutionary-conserved target for THs (38, 126); however, the presence of TRs in other types of testicular cells has been debated (8, 126). For example, both Leydig and Sertoli cells have been shown to express *tr*β in *D. rerio*; whereas *tr*α was only observed in Sertoli cells (38). In *R. norvegicus* testes, *tr*α mRNA was detected at all testis stages, while *tr*β could not be amplified at any of the stages studied (127, 128). Moreover, the fetal and prepubertal periods represent the highest expression of *trs* in mammals, predominantly *tr*α*1* (123), coinciding with high levels of *dio2* expression during these particular periods of testis development (116).

The expression of *trs* in testes is dependent on circulating TH concentrations. Recent studies in *S. iseri* and *R. norvegicus* demonstrated that *tr* mRNA levels fluctuate with TH production within gonadal tissues (117, 129). Moreover, the analysis of the promoter of TRα and TRβ showed putative thyroid response elements (TREs) in mice (*M. musculus*) and medaka (*Oryzias latipes*) (12), reinforcing the auto-regulation of TRs by THs. Also, it has been found that *tr*α and *tr*β transcript levels vary in testis tissue of the Brook trout (*Salvelinus fontinalis)* according to the seasons, with constant expression throughout spermatogenesis, and higher mRNA levels after spawning season (130). In addition, extra-thyroidal expression of TSH-receptors and TRH-receptors has been identified in the testes [*D. labrax* (131); fathead minnow, *Pimephales promelas* (132); Japanese quail, *Coturnix japonica* (133); *M. musculus*; *R. norvegicus*; Guinea pig, *Cavia porcellus*; and *O. aries* and *Homo sapiens* [reviewed in Ref. (125)]]. However, the regulatory role of TSH and TRH-receptors in the male gonad remains unclear.

Transmembrane transport of THs in the gonads is facilitated by the monocarboxylate transporter (Mct) family, specifically the solute carrier family 16 member 2 (Scl16a2 or Mct8) and the solute carrier family 16 member 10 (Scl16a10 or Mct10) (134–136). Muzzio et al. (137) found gender differences in transmembrane transporters, specifically *mct8*, in the gonads of the fathead minnow (*P. promelas*). The ovarian *mct8* mRNA levels were nearly twofold higher than testicular levels. However, *mct8* presented an antagonistic response with the goitrogen methimazole and T3 treatments. Similarly, in *P. promelas*, hypothyroid-induced condition up-regulates the expression of *mct8*; whereas hyperthyroidism condition decreases *mct8* transcripts (137). Therefore, it is important to include the regulation of the transmembrane proteins when studying the roles of THs in male reproduction.

## THs and Androgens in the Gonads

Thyroid hormones modulate androgen biosynthesis through direct and indirect regulation of the expression and activity of the steroidogenic enzymes involved in their synthesis [reviewed in-depth by Ref. (2, 6–12, 122)]. Recently, Flood et al. (12) performed an *in silico* analysis of the promoter of several enzymes and receptors involved in both the androgen and TH axes. It was found that several putative TREs and androgen responsive elements (AREs) were present in all of the androgen and TH-related genes studied. This reinforces the hypothesis of a potential direct crosstalk between these two endocrine axes and is supported by experimental approaches in several vertebrates. For example, in air-breathing catfish males (*Clarias gariepinus*), thiourea-treatment (TH inhibitor) led to selective down-regulation on the expression of the 11β-hydroxylase gene (*cyp11b1*) and 11β-hydroxysteroid dehydrogenase (*hsd11b2*); whereas no other alterations were observed for *3*β*-hsd*, *20*β*-hsd*, and *cyp17* (cytochrome P-450c17alpha) mRNA levels (40). In the same species, hypothyroidism-induction resulted in a reduction of 11-KT levels in serum and testis tissue (39). Moreover, in a *D. rerio* testis tissue culture, T3 alone stimulated the proliferation of both Sertoli cells and type A undifferentiated spermatogonia, resulting in newly formed spermatogonial cysts (38). However, T3 exposure alone produces no change in release of 11-KT; whereas when exposed to T3 in combination with FSH, a significant increase in 11-KT synthesis was observed (38). These results support the existence of a cross-regulation between THs (HPT axis) and androgens (HPG axis).

Thyroid hormone availability in the testes can be modulated at different levels of the HPG axis. Aforementioned, GnRH treatment increased TSH and T4 secretions in fish and amphibians (17, 33, 44, 45, 99); however, no changes in T3 were observed in *C. auratus* (35). These discrepancies in TH responses suggest that GnRH and gonadotropins can modulate the baseline of TH levels in plasma, but deiodinase activity would have to be stimulated in order to increase the concentration of the active T3. Thus, the expression of *dios* has been shown to respond to androgen signaling. Treatment with flutamide (an androgen receptor antagonist) produced a down-regulation of *tr*β in testes of *P. promelas* males (138). Additionally, androgens modulate TH synthesis and peripheral metabolism in fish. In *O. mykiss*, it was observed that T treatment had no effect on the plasma concentrations of T4, but reduced the levels of T3 (139). In tetrapods, androgen receptors (ar) have been identified in the thyroid gland of reptiles [American alligator, *Alligator mississippiensis*; (140)], and several mammals (141–143). These observations reinforce the idea that a direct crosstalk between HPG and HPT is possible.

## THs and Testicular Development

Thyroid hormones have considerable influence in the sexual ontogeny of male vertebrates, through direct interactions with genes involved in sex-determination and gonadal development in the HPG axis (12). It is known that THs play an important role in testicular development and function. In mammals, the genomic and non-genomic actions of THs during testicular development have been extensively reviewed (8, 10, 12). As described above, THs regulate proliferation and differentiation for both Sertoli and Leydig cells (104, 144). In rodent neonates, hypothyroidism and hyperthyroidism conditions affect the number of Sertoli cells by either extending or shortening their period of proliferation, respectively (145–149). Additionally in testes, TH-related machinery has distinct patterns of spatiotemporal expression with developmental stages. The expressions of *trs* and *dio2* decrease with gonadal maturation, suggesting that THs play a crucial role in early testis development and that cessation of TH signaling could be responsible for testis maturation [*O. mykiss* (114); *D. rerio* (150); *S. tropicalis* (83, 151); and *R. norvegicus* (121–123, 127, 152)]. Interestingly, *in situ* hybridization studies in *D. rerio* have shown that *dio1* and *dio2* mRNA levels were highest and concentrated at the rostral and caudal regions in the somite stages 6 through 18 (153), which are the stages at which gonadal development starts (154). The expression of *dio3* was first found in the 6-somite stage, with an increasing area and intensity through 22–24 h post-fertilization – the period at which sex differentiation occurs (153, 154). Altogether, these results demonstrate that maintenance of a baseline level of active T3 by deiodinases, as well as the TH machinery, could be necessary to vertebrate testis development.

In *D. rerio* testes, T3 in combination with FSH results in newly formed spermatogonial cysts and induces an increase in the synthesis of 11-KT (38). Moreover, it was observed in pejerrey fish (*Odontesthes bonariensis*), Japanese flounder (*Paralichthys olivaceus*), and *O. latipes* that environmental stressors, and/or cortisol treatment, induce 11-KT synthesis (25, 27, 28). It was suggested that the measured elevation of 11-KT could be explained through different mechanisms of action, including: the up-regulation of *hsd11b2* transcript [gene that codes for 11β-HSD; (84)], the inhibition of aromatase [enzyme that converts T to estradiol; (27)], and/or through the hepatic catabolism of cortisol (31, 155). Thus, the elevation of cortisol increases androgen biosynthesis with the concomitant masculinization of larvae (156). In summary, the crosstalk between HPA and HPG in the environmental sex-determination of fish has been heavily studied; however, due to the potential for interaction between HPT, HPA, and HPG axes, further studies are needed to clarify the role of the THs in the environmental sex-determination process.

## Conclusion

**Figure 3:**
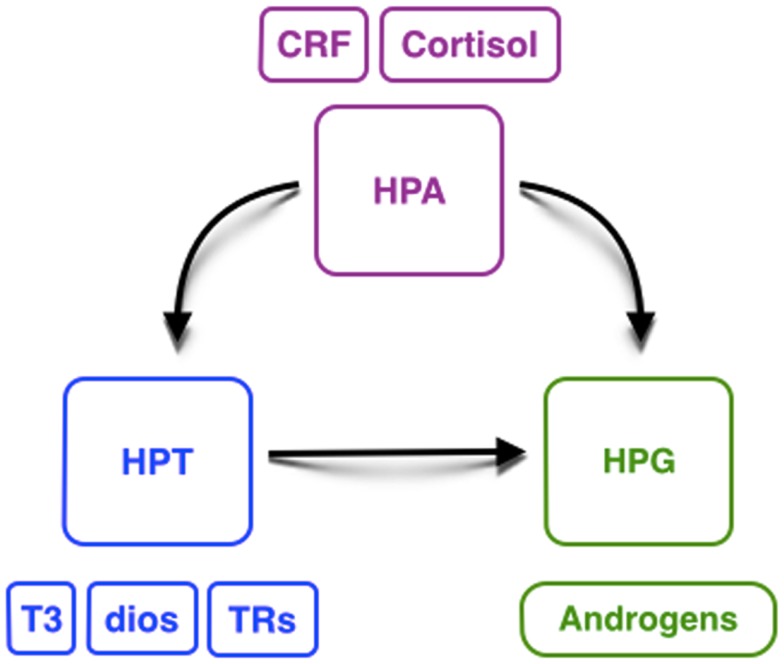
**Hypothetical interaction between the hypothalamic– pituitary–thyroid gland (HPT, blue), adrenal/interrenal (HPA, purple), and gonadal (HPG, green) axes**. CRF, corticotropin-releasing factor; T3, triiodothyronine; Dios, deiodinases; TRs, thyroid receptors.

This review on the interaction of HPT, HPA, and HPG axes illustrates our present understanding on the relationship between these endocrine axes and testicular development in different species of vertebrates, although it is necessary to confirm this hypothesis in other species (Figure 3). Some key points can be highlighted: (i) THs could have an important influence in gonadal development, especially on reproduction; (ii) there could be a relationship between T3, in combination with FSH, and induced androgen production, which is required to initiate spermatogenesis; (iii) the availability of deiodinases and TRs during testicular and early developmental stages could be crucial to exert TH action and to regulate testicular development; and (iv) the dual action of CRH on HPT and HPA axes could explain, at least in part, the high levels of androgens during the period of environmental sex-determination. Thus, we hypothesize that these hormonal axis interactions direct the gonadal fate toward masculinization.

## Conflict of Interest Statement

The authors declare that the research was conducted in the absence of any commercial or financial relationships that could be construed as a potential conflict of interest.
